# Species discrimination and total polyphenol prediction of porcini mushrooms by fourier transform mid‐infrared (FT‐MIR) spectrometry combined with multivariate statistical analysis

**DOI:** 10.1002/fsn3.1313

**Published:** 2020-01-14

**Authors:** Xiu‐Ping Li, Jieqing Li, Tao Li, Honggao Liu, Yuanzhong Wang

**Affiliations:** ^1^ College of Agronomy and Biotechnology Yunnan Agricultural University Kunming China; ^2^ Institute of Medicinal Plants Yunnan Academy of Agricultural Sciences Kunming China; ^3^ College of Resources and Environment Yuxi Normal University Yuxi China

**Keywords:** data fusion, FT‐MIR spectroscopy, porcini mushroom, species discrimination, total polyphenol prediction

## Abstract

The plateau specialty agricultural products, wild porcini mushrooms, have great value both as a superb cuisine and as a potential medication. Due to quality different between species added with the fraud behavior in sales process, make poor quality or poisonous sample inflow into the market, which pose a health risk for consumers, but also disrupted the mushroom market. Traditional analysis way is time‐consuming and laborious. Therefore, the aim of this study is to develop a way using fourier transform mid‐infrared (FT‐MIR) spectrometry and data fusion strategies for the fast and accurate species discrimination and predict amount of total polyphenol in four porcini mushrooms. The t‐distributed stochastic neighbor embedding based on mid‐level data fusion showed two species of *Boletus edulis* and *B. umbriniporus* have been identified. The order of correct rate of PLS‐DA models was mid‐level data fusion^q^ (100%) > mid‐level data fusion^e^ (97.06%) = mid‐level data fusion^v^ (97.06%) = stipes (97.06%) > low‐level data fusion (94.12%) > caps (91.18%). The order of correct rate of grid‐search support vector machine models was low‐level data fusion (100%) > caps (94.12%) > stipes (91.18%), and the order of particle swarm optimization support vector machine was low‐level data fusion (100%) > caps (97.06%) > stipes (88.24%). The mid‐level data fusion^q^ and low‐level data fusion had best discrimination accuracy (100%) allowing each mushroom classed into its real species, which could be used for accurate discrimination of samples. *B. edulis* mushrooms had highest total polyphenol, with 14.76 mg/g dw and 17.33 in caps and stipes mg/g dw, respectively. The phenols were easier to accumulate in the caps in *Leccinum rugosiceps* (1.03) and *B. tomentipes* (1.19), and the opposite phenomenon is observed in *B. edulis* (0.85) and *B. umbriniporus* (0.95). The correlation coefficient and residual predictive deviation of best prediction model were 86.76% and 2.40%, respectively, indicating that that there is good relevance between FT‐MIR and total polyphenol content, which could be used to predict roughly polyphenols content in mushrooms.

## Graphical abstract

The current study developed a way with fast, cheap and reliable for discriminating mushroom species and a way to roughly predict polyphenol content in mushrooms. Besides, the study applied three ways of feature extraction and provided data of content and enrich ability of total polyphenol in different morphological part of mushroom for the first time.

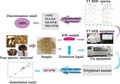

## 1 INTRODUCTION

Mushrooms existed on earth before human appeared (Bilal, Ahmad, & Wani, 2010) and have been consumed over a long period of time (Manzi, Gambelli, Marconi, Vivanti, & Pizzoferrato, 1999). The edible porcini mushrooms as a kind of macro‐fungus belong to *Boletus* and *Leccinum* genus is large enough to be picked and ate valued in gourmet. These mushrooms are known as reputation of “vegetable meat” with characteristics of hypertrophic fruiting body, pleasant aroma, and unique taste. Besides, they have other features of high in proteins while low in fat and calories (Manzi et al., 1999). The porcini mushrooms contain natural chemical compositions such as proteins, phenols, flavones carbohydrates, alkaloids, vitamins, minerals, which are useful in nutrition and medicine purposes like antioxidant, antiviral, anti‐tumor, antifungal, and enhance immunity (Bilal et al., 2010; Kaewnarin, Suwannarach, Kumla, & Lumyong, 2016). They have also many mineral compounds including valued selenium with high antioxidant properties, but it can be dependent on species and its place of origin (Bekyarov et al., 2011). Polyphenols are one of the compound which have positive function for human health (Wong & Chye, 2009), wildly finding in vegetables, fruits, and grain products (Velioglu, Mazza, Gao, & Oomah, 1998). Studies have revealed that polyphenols available from mushrooms have higher antioxidant activities compared with many fruits and vegetables (Ismail, Marjan, & Foong, 2^2004^; Jia, Tang, & Wu, 1999; Velioglu et al., 1998).

The total porcini mushrooms in Yunnan Province are approximately to be 224 species in which 114 species are edible. Porcini mushrooms are one of the “four kings of mushroom” and are one of the traditional bulks exported agricultural products. According to statistical data, export quantity of wild porcini mushrooms from Yunnan Province accounts for about 80% of total export quantity of China (http://ynsyj.yncoop.com). The average price per kilogram of hot‐selling porcini mushrooms can reach about 90 CNY according to market investigation at August 2019. Due to high economic value, mushroom can be used by people of noncompliance with the morals to seek profits. Some fraud actions have been reported. Several mushrooms without formally scientific names have been sold with the mushrooms from species of *porcini* (Dentinger & Suz, 2014). The fraudulent action is also reported by Casale et al. (2012), which revealed that commercial dried mushrooms which are composed of *Boletus edulis* and related species have been adulterated with *B. violaceofuscus*. These actions have seriously influencing mushrooms quality and interfering quality supervision, resource evaluation, food safety, and even man health. In addition, there is just one specie namely *Phlebopus portentosus* can be cultivated successfully, and the over‐picking by local peoples of lower income makes the seasonal wild porcini mushroom increasingly rarer. These reasons stated above confirm the urgent necessity regarding the species discrimination for porcini mushrooms.

Morphology is a common method based on surface features from fruiting body such as color, shape, size, or reticulate pattern (Tsujikawa et al., 2003). However, this method did neglect the phenotypic variability of mushroom. Besides, fruiting body will lose its characteristics when it suffered from drying, dehydration, salting, and bleaching, and usually processed into slice, can, or extractum to adapt long‐distance transportation for exporting. Molecular tools have been developed to elude these flaws. Internal transcribed spacer (ITS) primer of 28 *B. edulis* samples combining phylogenetic tree analysis has provided an effective identification and revealed that *B. edulis* mushrooms are often sold by intermixed with mushrooms species of *B. violaceofuscus* (Mello et al., 2006). Another study for species clarification used ITS and fungal immunomodulatory protein (FIP) sequences (Zhou, Liu, Guo, Su, & Zhang, 2016), shown that mushrooms from *Ganoderma* actually belong to different species. Now, nevertheless, infrared spectroscopy is preferred by researchers because this analytical technique has advantages of wide range of applications (liquid, solid, and gaseous samples), less sample consumption, without destroying samples and rapid, not only for compound identification and molecular structure characterization, but also for quantitative analysis (Borràs et al., 2015; Dutta, 2017). Just as there are no two identical leaves in the world, there are also differences between samples, which can be displayed in the infrared spectrum. The mushroom specie of *Catathelasma ventricosum* had higher amounts both of total phenols (9.24 ± 0.42 mg/g dw) and total tocopherols (2.76 ± 0.22μg/g dw) than species of *Clitocybe maxima* (6.96 ± 0.32 mg/g dw and 0.62 ± 0.02 μg/g dw), *Stropharia rugoso‐annulata* (5.52 ± 0.45 mg/g dw and 1.34 ± 0.17 μg/g dw), *Craterellus cornucopioides* (5.39 ± 0.28 mg/g dw and 1.94 ± 0.43 μg/g dw), and *Laccaria Amethystea* (9.08 ± 0.54 mg/g dw and 2.72 ± 0.27 μg/g dw) (Liu et al., 2012). Therefore, the *C. ventricosum* species may have higher power to decrease the oxygen radicals (Kim et al., 2008). Influenced by latitude, the sea buckthorn berries collected from Québec, Canada, may have better pleasant taste than those grown at Sammalmäki, Finland, which contributed by higher levels of total sugar and sugar/acid ratio and a lower level of total acid (Zheng, Yang, Trépanier, & Kallio, 2012). It is this variability between samples that is key for building spectroscopic fingerprint spectrum and make it possible to track the sample source. Fingerprint spectrum available from Fourier transform mid‐infrared (FT‐MIR) spectra has been united with chemometrics for food quality like tannin characterization (Tondi & Petutschnigg, 2015), honey adulteration (Das et al., 2017), camel milk metamorphism (Nagy et al., 2019), content determination of caffeine and trigonelline (Hagos, Redi‐Abshiro, & Chandravanshi, 2018), variant screen of short‐chain fructooligosaccharides (scFOS) (Trollope, Nieuwoudt, Görgens, & Volschenk, 2014), and storage effects on saffron (Ordoudi, de los Mozos Pascual, & Tsimidou, 2014). Despite the excellent practicability of FT‐MIR spectroscopy, potential flaw of the single data matrix is that it is not enough to represent the entire sample (Borràs et al., 2015).

Data fusion is a rising technology intersected by multiple disciplines for resulting in a satisfactory inference. Thanks to the rapid development of computers, improvement of experimental instruments, and gradual maturation of data fusion; data fusion has been broadly used in many areas including environment supervision, food quality, medical diagnosis, military, mapping, and robot (Hall & Llinas, 2001). Low‐level data fusion is an effective strategy to distinguish official rhubarb (recorded in the Chinese Pharmacopoeia) and unofficial rhubarb (Sun, Zhang, Zhang, & Zhu, 2017), and also to trace geographical origin of herbal medicine of *Panax notoginseng* (Li, Zhang, & Wang, 2018). Another commonly used strategy is mid‐level data fusion. Unlike direct data splices in low‐level data fusion, mid‐level data fusion digs effective information available from singe data matrices in order to enhance run speed of algorithm and output a better outcome. This strategy of mid‐level data fusion has been used to trace specie and geographical origin of Porcini mushrooms (Yao, Li, Liu, Li, & Wang, 2018) and also to classify organic and nonorganic orange juices (Cuevas, Pereira‐Caro, Moreno‐Rojas, Muñoz‐Redondo, & Ruiz‐Moreno, 2017), and the manufacturer of beer with same brand and product could be discriminated (Vera et al., 2011). According to literatures, the several common ways of feature selection are: as follows (1) latent variables (LVs) which selected according to R^2^Y_(cum)_ and Q^2^_(cum)_ based on partial least squares‐discriminant analysis (PLS‐DA) model (Yao et al., 2018), (2) principal components (PCs) obtained from principal component analysis (PCA) (Vera et al., 2011), (3) variable importance in the projections (VIPs) picked by the values of VIP >1 (Qi, Liu, Li, Li, & &. Wang, 2^2018^). R^2^Y_(cum)_ and Q^2^_(cum)_ were used to assess the ability of the model to fit data of training set and to predict new sample (test set). The R^2^Y_(cum)_ represents the degree of fitting the data; a large value (close to 1) is usually a necessary condition for a good model. However, the Q^2^_(cum)_ represents the predictivity of model, a large value (>0.5) indicates good potential to predict sample origin. VIP values larger than 1 indicate “important” X‐variables, while the values lower than 0.5 indicate “unimportant” X‐variables.

Although there are many studies on the species discrimination using data fusion strategy, there is no enough information regarding the data fusion using different morphological parts of porcini mushrooms of Yunnan province. The current study aims to find a fast and simple way to discriminate species and predict content of polyphenol using Fourier transform mid‐infrared (FT‐MIR) spectra available from different morphological parts of mushroom.

## 2 METHODS

### 2.1 Sampling and sample preparation

A total of 100 fruiting bodies of the four different species (*Boletus edulis* Bull. Fr, *Leccinum rugosicepes* (Peck) Sing, *B. tomentipes* Earle and *B. umbriniporus* Hongo) were collected from Yuxi, Yunnan province, China, during 2011–2012. The detailed information both of samples and collected place is shown in Table 1. Picking behaviors were carried out in the forests. In other words, the collection locations were in mountain area, which is far away from villages and human activities. Therefore, these mushrooms could be considered as clean and undamaged. Fruiting bodies of similar‐sized and robust were selected for analysis.

**Table 1:** The information of mushrooms samples

Code	Species	Location	Quantity
1	*Boletus edulis* Bull. Fr	Pubei, Yimen, Yuxi	11
2	*B*. *edulis* Bull. Fr	Pubei, Yimen, Yuxi	7
3	*B*. *edulis* Bull. Fr	Pubei, Yimen, Yuxi	10
4	*B*. *edulis* Bull. Fr	Pubei, Yimen, Yuxi	10
5	*Leccinum rugosicepes* (Peck) Sing	Pubei, Yimen, Yuxi	9
6	*L*. *rugosicepes* (Peck) Sing	Pubei, Yimen, Yuxi	7
7	*B*. *tomentipes* Earle	Fuliangpeng, Eshan, Yuxi	6
8	*B*. *tomentipes* Earle	Xiaojie, Eshan, Yuxi	9
9	*B*. *tomentipes* Earle	Tongchang, Yimen, Yuxi	10
10	*B*. *tomentipes* Earle	Chah, Eshan, Yuxi	7
11	*B*. *umbriniporus* Hongo	Huangcaoba, Yuxi	8
12	*B*. *umbriniporus* Hongo	Tongchang, Yimen, Yuxi	6

The fresh fruiting bodies were treated using soft brush to cast off soils and other litters (leaves or branches) and then washed with running tap water until no impurity is visible by naked eyes. These watery mushrooms were dried in laboratory oven at 50°C for constant mass and crushed immediately. It is underlined that each dried fruiting body was crushed after dividing into two morphological parts, namely cap and stipe. Finally, the crushed sample was passed through an 80‐mesh sieve and then stored in zip lock bag wait for next analysis.

### 2.2 Determining FT‐MIR information

Spectra information was determined using common method of KBr tableting. The 1.0 ± 0.2 mg sample was homogenized using 100 ± 0.2 mg KBr powder in an agate mortar in first step. Because KBr is easy to absorb moisture, this step was completed under infrared light. After thoroughly homogenization, mixed sample was adjusted to a thin slice under pressure available from tablet press. In last step, the FT‐MIR spectra were immediately determined with a Fourier transform infrared spectroscopy spectrometer equipped with a DTGS detector. The determination conditions of wavenumber range, the number of successive scans, and resolution were set as 4,000 to 400 cm^−1^, 64 and 4 cm^−1^, respectively.

### 2.3 Determining amount of total polyphenol

The unerring 0.2500 g sample was extracted by an ultrasonic cleaning machine with 5 ml (solid‐liquid ratio of 1:20) of 40% ethanol at room temperature for 30 min. Then, the extracted liquid was filtered through qualitative filter paper, and the filtrate was adjusted to 25 ml with deionized water.

The determination method of vis absorbance was similar to the procedures reported by Kaewnarin et al. (2016). The 0.1 ml filtrate was transferred in a test tube using transfer liquid gun and then added into 7.9 ml distilled water to dilute 80 folds. After that, the sample was added with 0.5 ml of 1 mol/L Foline–Ciocalteu reagent. Eight minutes later, 1.5 ml of 20% saturated sodium carbonate solution was added to the diluted sample. After seal by preservative film, the sample was incubated for 1 hr at room temperature and then determined using visible spectrophotometer at 760 nm. Calibration curve for quantification was constructed by gallic acid. All the reagents belong to analytical reagent (AR) grade.

### 2.4 Statistical analyses

Spectra information was converted to data matrix using software Omnic 8.0 (Thermo Fisher Scientific Inc.). In order to fuse low‐level data, data matrices from the two parts were directly spliced. Three types of feature variables were used, which correspond to PCs, LVs, and VIPs. And the selection standards were eigenvalue >1, maximum Q^2^_(cum),_ and VIP >1, respectively. These feature matrices were spliced according to same extraction way to get three new data matrices for mid‐level data fusion. The t‐distributed stochastic neighbor embedding (t‐SNE) was applied to preliminarily visualize discrimination of mushrooms. For building discrimination model, fix data matrices (two single, one low‐level data fusion and three mid‐level data fusion) were divided into training set and test set according to Kennard–Stone (KS) algorithm. Training set with 2/3 data was used to build model, while the rest of data namely test set were used to test discriminatory capacity of the model. Supervised classification methods of PLS‐DA, grid‐search support vector machine (GS‐SVM) and particle swarm optimization support vector machine (PSO‐SVM) were developed with help of software of SIMCA‐P^+^ 13.0 (Umetrics AB) and MATLAB R2014a (Math works).

In essay of total polyphenol prediction, the amount and extraction rate of total polyphenol of per sample were calculated based on vis absorbance. Then, the data of amount and FT‐MIR both from same morphological part were spliced to develop two new data matrices for total polyphenol prediction. Data treatments of first‐order derivative (FD), second‐order derivative (*SD*), multiplicative scattering correction (MSC), standard normal variate (SNV), Savitzky–Golay (SG) smoothing, and combination of these treatments were performed to optimize spectral data. The derivative order, points in each, and distance between each were set as 1, 15, and 1 and 2, 15, and 1 in FD and FD, respectively. The order derivative was powerful to enhance resolution, whereas it also enhanced the noise (Roy, 2015). SG smoothing may be a helpful method to solve this problem (Xu et al., 2008). The MSC and SNV were usually applied to reduce the light scatter effect caused by sizes and shapes of granular sample (Helland, Naes, & Isaksson, 1995). Before KS, data matrices were normalized to a range of 1–2 to avoid reduced accuracy brought by different dimensions. The method of KS was as described above. The prediction of total polyphenol was performed using supervised regression methods of GS‐SVM. Root mean square error of cross validation (RMSECV) and root mean square error of prediction (RMSEP) were applied to estimate total error for samples and estimate of total prediction error, respectively. The calculation approaches were as follows:

$$\text{RMSECV}=\sqrt{\frac{\sum_{i=1}^{n}{\left({\hat{y}}_{i}-y_{i}\right)}^{2}}{n_{c}}}$$

$$\text{RMSEP}=\sqrt{\frac{\sum_{i\;=\;\text{1}}^{n_{P}}{\left({\hat{y}}_{i}-y_{i}\right)}^{2}}{n_{P}}}$$

where the n_c_ represents the quantity of samples of training set while n_p_ represents the quantity of samples of test set, and the ${\hat{y}}_{i}$ and y_i_ represent the predicted and measured value, respectively.

Coefficient of determination (R^2^) was used to reflect the correlation relationship between predicted and measured value. Another useful assessment parameter of residual predictive deviation (RPD) was calculated via standard deviation (*SD*) divide by RMSEP. The calculation methods were as follows:

$$R^{2}=\frac{\sum_{i=1}^{n}{\left({\hat{y}}_{i}-y_{i}\right)}^{2}}{\sum_{i=1}^{n}{\left({\hat{y}}_{i}-y\right)}^{2}}$$

$$\text{RPD}=\frac{\text{SD}}{\text{RMSEP}}$$

**Figure 1:**
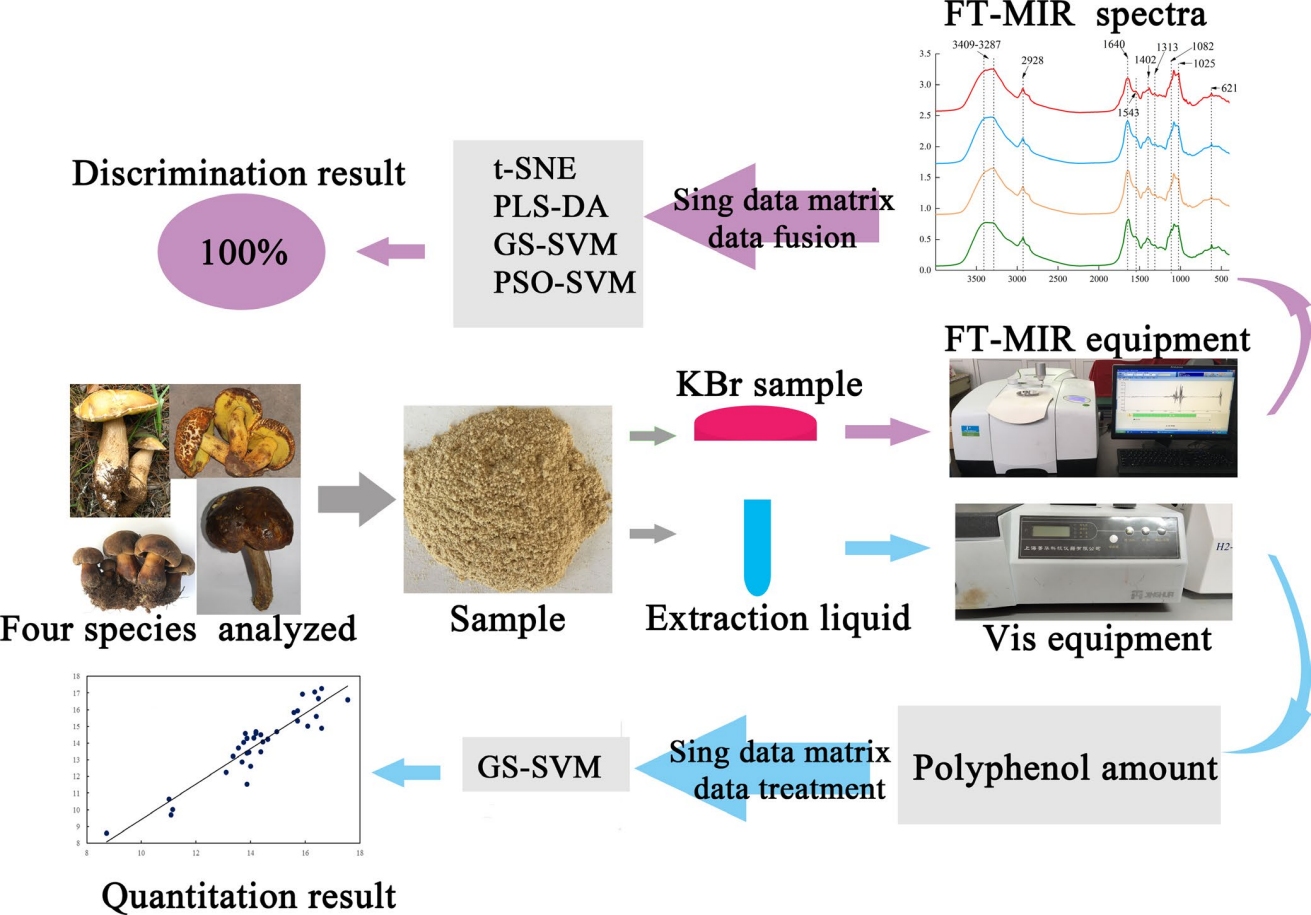
Workflow of species discrimination and total polyphenol prediction

The RPD value between 1.5 and 2 indicated that the model can discriminate low values from high values of the contents. For predicting roughly, the RPD between 2 and 2.5 is a necessary condition, while the RPD greater than 2.5 or 3 corresponds excellent power for content prediction (Nicolaï et al., 2007). It should be noted that the data of amounts and spectra were one‐to‐one correspondence in total polyphenol prediction. For better understanding this study, the workflow is shown in Figure 1.

## 3 RESULTS

### 3.1 FT‐MIR absorption peaks interpretation

**Figure 2:**
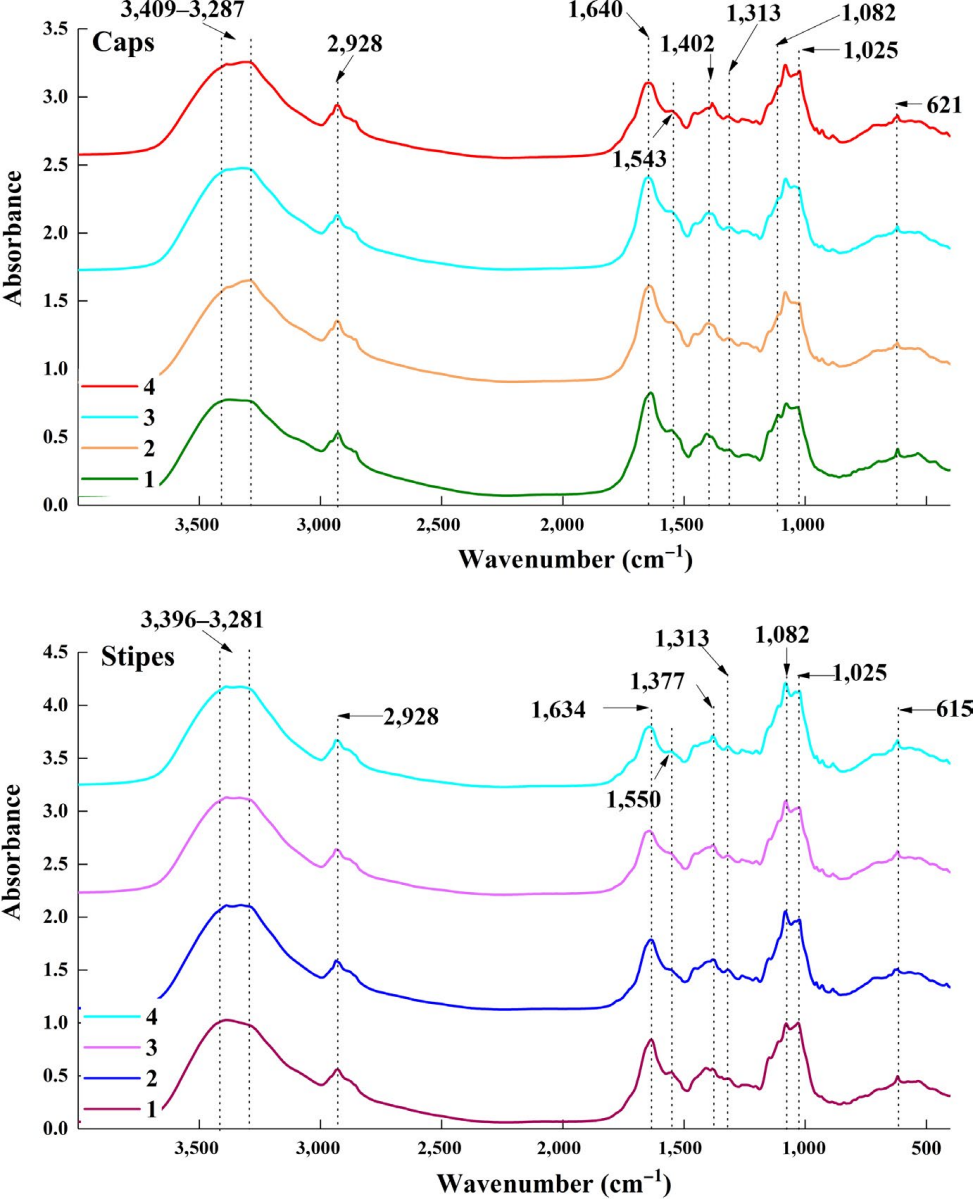
Average FT‐MIR spectra of the caps and stipes. 1, *B. edulis*; 2, *L. rugosiceps*; 3, *B.  omentipes*; 4, *B. umbriniporus*

The raw average spectra of caps and stipes from four species of porcini mushrooms are shown in Figure 2. The major absorption peaks of gaps and stipes have been interpreted. It can be seen from Figure 2 that the absorption bands of 2,928, 1,313, 1,082, and 1,025 cm^−1^ were shared in caps and stipes. The single perk at 2,928 cm^−1^ could be assigned to methylene C‐H asymmetric stretching; the weak peak around 1,313 cm^−1^ was the vinylidene C‐H in‐plane bend (Coates, 2000). The peaks around 1025–1082 cm^−1^ corresponded to ‐C‐O stretching (Yang & Irudayaraj, 2000). Two broad peaks of 3409–3287 and 3396–3281 cm^−1^ were assigned to the N‐H stretching vibration (Kasprzyk, Depciuch, Grabek‐Lejko, & Parlinska‐Wojtan, 2018). The around peaks at 1,640, 1,634, 1,543, and 1,550 cm^−1^ belonged to the C=O stretching, C‐N stretching, and N‐H bending vibration in amide‐I and in amide‐II bands (Choong et al., 2011), which mean these mushrooms contain protein. The peaks of 1,402 and 1,377 cm^−1^ were O‐C‐H bending and C‐H bending, respectively (Chen, Guo, Yan, Sun, & Zhou, 2017). Although values of absorption peaks were varied in caps and stipes, the shapes and positions of the absorption peaks were similar among all samples, which revealed that the substance components are similar within mushrooms of four species.

### 3.2 Visualizing discrimination by t‐SNE

PCA and t‐SNE are the most common approaches to provide more vivid and easy‐to‐understand classification result. However, both methods can be used to draw two‐ or three‐dimensional graphics to visualize sample classification. The difference is that the t‐SNE can capture more information to draw the graphics using random walks on neighborhood graphs. Studies have reported that t‐SNE overmatches existing most advanced techniques such as sammon mapping, lsomap, or locally linear embedding (Maaten & Hinton, 2008).

**Figure 3:**
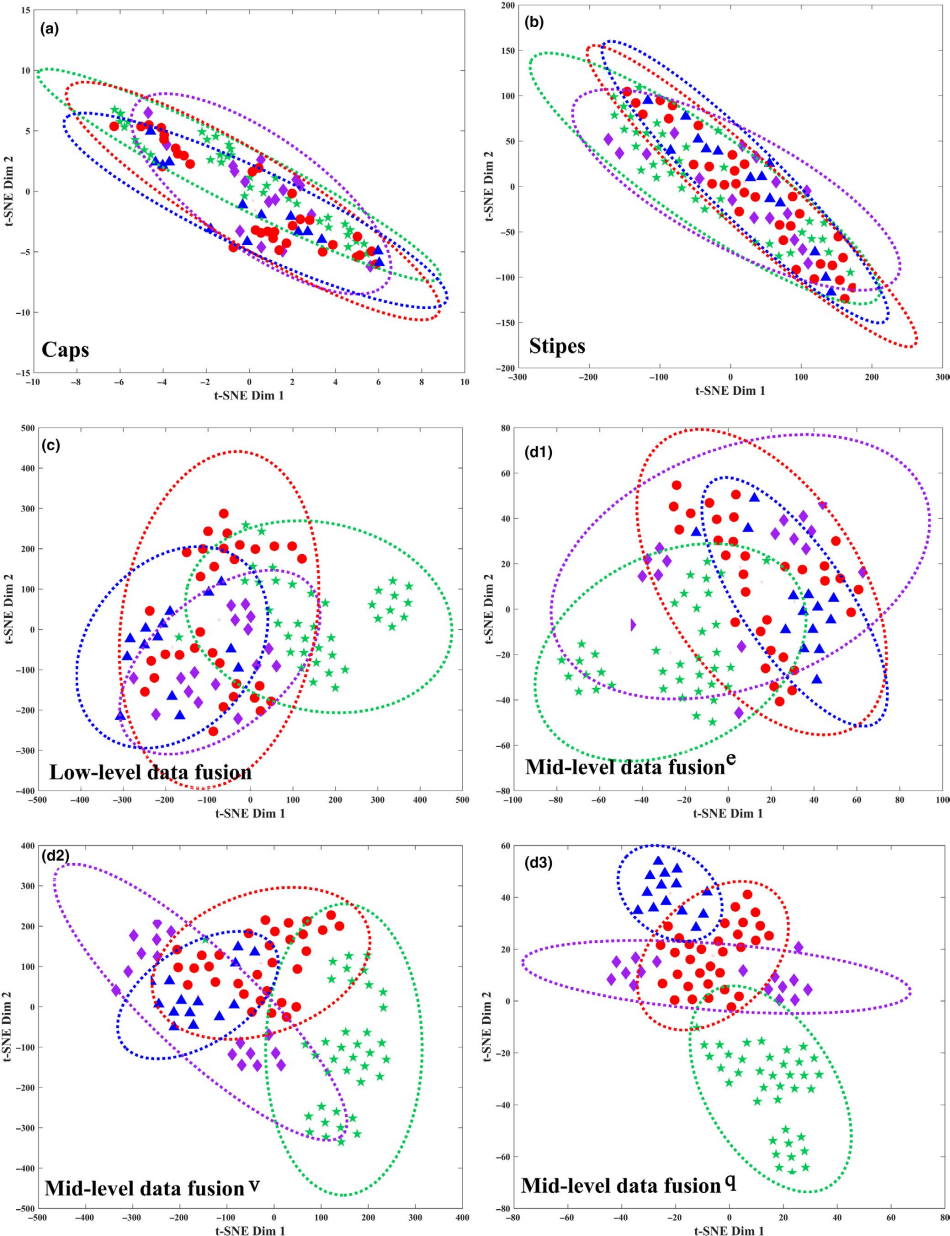
Mushroom discrimination by t‐SNE. 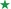, *B. edulis*; 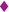, *L. rugosiceps*; 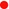, *B. tomentipes*; 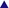, *B. umbriniporus*; e, eigenvalue selection by eigenvalue >1; v, variable selection by VIP >1; q, variable selection by maximum Q^2^

Here, specie discrimination by t‐SNE is shown in Figure 3 where dotted circle represents 95% confidence level of per species. Mushroom classifications were disordered using single data matrices in which any one species could not be separated (Figure 3a,b). Low‐level data fusion was superior to single data matrix (Figure 3c). Samples show clustering tendency, and the samples that belong to species of *B. edulis* show an obvious clustering, although one sample is beyond confidence interval and far away from other samples within the species. Mid‐level data fusion^e^ shows a better result, with all samples that belong to *B. edulis* clustered together and landed within confidence interval but still be mixed with samples belonging to other species (Figure 3d1). Compared with mid‐level data fusion^e^, mid‐level data fusion^v^ displayed a better discrimination in which one species namely *B. eduli*s was separated completely (Figure 3d2). The mid‐level data fusion^q^ had best cluster classification among all scatterplots, which allowed two species of *B. eduli*s and *B. umbriniporus* be discriminated (Figure 3d3). Besides, clustering results reveal that the within a species is smaller than that between species since the clustering effect was better in same species than in different species. For higher discrimination result which per sample was classified into actual species, supervised model combination with data fusion was performed.

### 3.3 Discriminating samples by PLS‐DA model

The PLS‐DA is confirmed as a universal strategy for reducing variables, regression prediction, source discrimination, etc. (Loong, Liong, & Jemain, 2018). Table 2 shows the results both the parameters of models and discrimination accuracy of test set. The parameters of RMSEE and RMSECV were used to assess the reliability of training set and test set, respectively; a small value is usually necessary for model quality. Compared with caps, the stipes model has better performance, with the values of parameters of R^2^Y_(cum)_, Q^2^_(cum)_, RMSEE, and RMSECV as 0.9, 0.7, 0.15, and 0.26, respectively, and the total correctness rate of test set, with a rate of 97.06%, was higher than that of caps, with a rate of 91.18%. In data fusion strategies, the same total accuracy of 97.06% has been noticed in mid‐level^e^ and mid‐level^v^ data fusion. Two samples from species of *L. rugosiceps* were misclassed into *B. tomentipes,* both in mid‐level^e^ and mid‐level^v^ data fusion. The lower accuracy (94.12%) at low‐level data fusion may due to redundant information brought by data direct splicing (Borràs et al., 2015). Importantly, model of mid‐level data fusion^q^ has highest value of R^2^Y_(cum)_ (0.91), Q^2^_(cum)_ (0.86) and lowest value of RMSEE (0.13), RMSECV (0.19), which meant that the model could fit the new data very well and had a good potential to predict sample origin, and the fact is that this model did allow per sample classed into real species corresponding to total accuracy of 100%. The order of accuracies in all classification models is as follows: mid‐level data fusion^q^ > mid‐level^e^ data fusion = mid‐level^v^ data fusion = stipes > low‐level data fusion > caps, which indicated that mid‐level data fusion^q^ combined with PLS‐DA model can be seen as a reliable way to discriminate mushroom species.

**Table 2:** Mushroom discrimination by PLS‐DA

Caps						
		R^2^Y_(cum)_	Q^2^_(cum)_	RMSEE _(avg)_	RMSECV _(avg)_	LV_S_
		0.84	0.63	0.18	0.26	12
		1^a^	2	3	4	Accuracy (%)
	1^b^	14	0	0	0	100
	2	0	3	2	1	50
	3	0	0	10	0	100
	4	0	0	0	4	100
Total	34	14	3	12	5	91.18

Stipes						
		R^2^Y_(cum)_	Q^2^_(cum)_	RMSEE _(avg)_	RMSECV _(avg)_	LV_S_
		0.90	0.70	0.15	0.26	14
		1^a^	2	3	4	Accuracy (%)
	1^b^	14	0	0	0	100
	2	0	5	1	0	83.33
	3	0	0	10	0	100
	4	0	0	0	4	100
Total	34	14	5	11	4	97.06

Low‐level data fusion						
		R^2^Y_(cum)_	Q^2^_(cum)_	RMSEE _(avg)_	RMSECV _(avg)_	LV_S_
		0.87	0.69	0.15	0.24	8
		1^a^	2	3	4	Accuracy (%)
	1^b^	14	0	0	0	100
	2	0	4	1	1	66.67
	3	0	0	10	0	100
	4	0	0	0	4	100
Total	34	14	4	11	5	94.12

Mid‐level data fusion^e^						
		R^2^Y_(cum)_	Q^2^_(cum)_	RMSEE _(avg)_	RMSECV _(avg)_	LV_S_
		0.87	0.79	0.15	0.21	4
		1^a^	2	3	4	Accuracy (%)
	1^b^	14	0	0	0	100
	2	0	5	1	0	83.33
	3	0	0	10	0	100
	4	0	0	0	4	100
Total	34	14	5	11	4	97.06

Mid‐level data fusion^v^						
		R^2^Y_(cum)_	Q^2^_(cum)_	RMSEE _(avg)_	RMSECV _(avg)_	LV_S_
		0.84	0.56	0.17	0.28	7
		1^a^	2	3	4	Accuracy (%)
	1^b^	14	0	0	0	100
	2	0	5	0	1	100
	3	0	0	10	0	100
	4	0	0	0	4	83.33
Total	34	14	6	10	5	97.06

Mid‐level data fusion^q^						
		R^2^Y_(cum)_	Q^2^_(cum)_	RMSEE _(avg)_	RMSECV _(avg)_	LV_S_
		0.91	0.86	0.13	0.192761	3
		1^a^	2	3	4	Accuracy (%)
	1^b^	14	0	0	0	100
	2	0	6	0	0	100
	3	0	0	10	0	100
	4	0	0	0	4	100
Total	34	14	6	10	4	100

Note

1, *B*. *edulis*; 2, *L. rugosiceps*; 3, *B*. *tomentipes*; 4, *B*. *umbriniporus*; FD, first‐order derivative, LVs: the number of potential variables.

a Predicted category.

b Genuine category.

### 3.4 Discriminating samples by SVM model

Supervised models of GS‐SVM and PSO‐SVM were developed to compare with the models of PLS‐DA for fast and simple way for discriminating mushroom species. Table 3 summarizes the results of both the parameters of model and total correctness rate. As shown in Table 3, the model PSO‐SVM had higher accuracy of test set than GS‐SVM based on caps data, with 97.06% higher than 94.12%, and the best parameters of penalty parameter (c) and kernel parameter (g) were 30.44 and 20.93, respectively. Correspondingly, the misclassified samples of PSO‐SVM were lower than GS‐SVM, with one lower than two. In the models of stipes, however, discrimination accuracy was lower in PSO‐SVM model (88.24%) compared with GS‐SVM model (91.18%). The highest accuracy was noticed using low‐level data fusion strategy, which means the 100% correctness rate and without misclassified sample in both GS‐SVM and PSO‐SVM. Regarding the best discrimination model, low‐level data fusion combined with model of GS‐SVM or PSO‐SVM could be used as a fast and simple approach to discriminate mushroom species.

**Table 3:** Mushroom discrimination by GS‐SVM and PSO‐SVM

Origin of data	Strategy	Best c	Best g	Accuracy of training set (%)	Accuracy of test set (%)
Caps	GS‐SVM	1.05e + 06	1.91e−06	87.88	94.12 (32/34)
	PSO‐SVM	30.44	20.93	89.39	97.06 (33/34)
Stipes	GS‐SVM	2,896.31	6.91e−04	93.94	91.18 (31/34)
	PSO‐SVM	2.38	54.73	92.42	88.24 (30/34)
Low‐level data fusion	GS‐SVM	5.66	45.25	92.42	100 (34/34)
	PSO‐SVM	7.9	27.85	92.42	100 (34/34)

### 3.5 Total polyphenol content

In this study, 40% ethanol was used for total polyphenol extraction from mushroom. The arithmetic means, standard deviation, median value, content range, extraction percentage, and bioconcentration factor of phenolic compounds in two morphological parts of mushrooms are presented in Table 4.

**Table 4:** The arithmetic means, standard deviation, median value, amount range, extraction percentage, and bioconcentration factor of total phenolic content in two morphological parts of mushrooms

Morphological part	Species			
	*B. edulis* *n* = 39_C_ *n* = 38s	*L. rugosiceps* *n* = 17 *n* = 16	*B*. *tomentipes* *n* = 43 *n* = 42	*B*. *umbriniporus* *n* = 35 *n* = 37
Caps	15.10 ± 1.37	14.33 ± 2.02	12.27 ± 1.85	10.22 ± 1.97
	14.76	14.06	12.03	10.13
	12.28–17.55	9.49–17.04	8.98–16.34	7.59–16.91
	1.51	1.4	1.23	1.02
Stipes	15.81 ± 3.48	14.00 ± 2.10	11.30 ± 3.83	10.74 ± 1.40
	17.33	13.68	10.09	10.70
	8.16–19.71	10.63–19.13	6.32–18.50	7.59–13.42
	1.58	1.4	1.13	1.07
BCFc‐s	0.85	1.03	1.19	0.95

Abbreviations: dw, dry weight; C, the number of caps; s, the number of stipes; BCFc‐s, quotient calculated by the median value of cap divided by the median value of stipe.

The median values of total phenolic content for four species analyzed varied between 10.13 and 14.76 mg/g dw in caps, and between 10.70 and 17.33 mg/g dw in stipes. Compared with *B. tomentipes*, *L. rugosiceps* have greater total polyphenol content, with the median values of the caps and stipes as 14.6 mg/g dw and 13.68 mg/g dw, respectively, then 12.03 mg/g dw and 10.09 mg/g dw, respectively. The smallest contents were found in species of *B. umbriniporus*, with the median values of 10.13 mg/g dw in caps and 10.70 mg/g dw in stipes. Among these results, mushrooms belong to *B. edulis* showed greatest total phenolic content both among the caps (14.76 mg/g dw) and stipes (17.33 mg/g dw). The content range of caps was from 12.28 to 17.55 mg/g dw, while the range of stipes was from 8.16 to 19.71 mg/g dw. Higher total phenolic amount may be the major contributes to the better flavor of mushrooms of *B. edulis* which show higher popularity in customers.

Table 4 also shows the value of bioconcentration factor (BCF) in order to assess enrichment ability of total polyphenol by different morphological parts of mushroom for the first time. The BCF was calculated by the median value of cap divided by the median value of stipe. The enrichment ability of total polyphenol varied from 0.85 to 1.19, with the values of 0.85 for *B. edulis*, 0.95 for *B. umbriniporus*, 1.03 for *L. rugosiceps,* and 1.19 for *B. tomentipes*. Apparently, caps were considered as a morphological part to better accumulate total polyphenol compared with stipes in species of *L. rugosiceps* and *B. tomentipes*, while contrary results were noticed in *B. edulis* and *B. umbriniporus*. In a previous study, the mushroom caps of species of *Agrocybe aegerita* and *Lentinula edodes* have higher content of glutathione, ergothioneine than in stipes (Kalaras, Richie, Calcagnotto, & Beelman, 2017). One possible explanation is that the mushroom cap consisted of pileipellis, flesh, and hymenium, which are important for its metabolism and reproduction (Casale et al., 2012). Therefore, cap of mushroom may be a major part to enrich active constituents and can be the primary choice to meet purpose of diet nutrition and natural antioxidants.

### 3.6 Predicting content of total polyphenol

**Figure 4:**
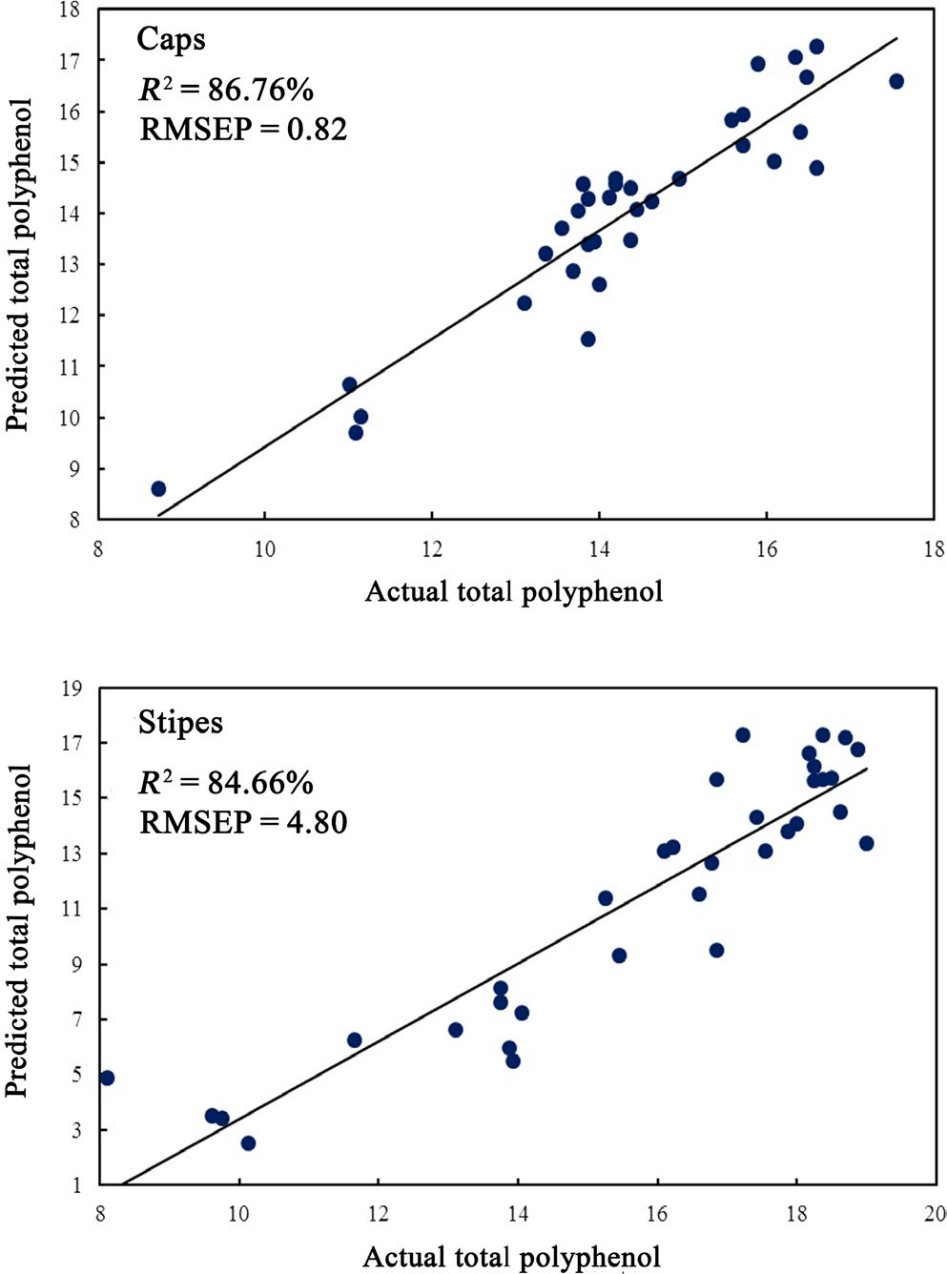
Best prediction results of total polyphenol

The regression model of GS‐SVM was developed using raw spectral information from two morphological parts of mushrooms combined with various data treatments to predict amount of total polyphenol in four porcini mushrooms. Table 5 shows the results, and the best prediction results by the two parts are shown in Figure 4. The RPD value of models based on four data treatments of FD, FD + SG (7), FD + SG (9), and SNV + FD+SG (9), respectively, was 1.52, 1.51, 1.50, and 1.86 when using caps data, which indicated these models could be used to distinguish sample with high concentration of polyphenol. The best prediction model was achieved by *SD*‐treated, the RMSEP, RPD and R^2^ were 0.82, 2.4 and 86.76%, which indicated the good relationship between polyphenol content and FT‐MIR data, and this model could be applied to predict total polyphenol content roughly. In models by stipes data, the best R^2^ (84.66%) was obtained by FD‐treated, which indicated the good relationship between polyphenol content and FT‐MIR data. However, the best prediction was achieved by model with data treatment of MSC + FD+SG (9), with the values of 0.83, 2.32, and 1.78 corresponded to R^2^, RMSEP, and RPD, respectively, which indicated that the model could discriminate high values from low values among these amounts. In summary, the prediction result by caps data was superior to result by stipes data. Although numerous data treatments have been used to improve data quality, this study did not obtain an excellent model for accurately predicting the content of total polyphenol. This result may be caused by insufficient sample size, and better model may be obtained by increasing sample size and applying data fusion strategies in the future.

**Table 5:** Prediction results of total polyphenol by GS‐SVM regression model

Substrate	Treatent	Best c	Best g	Cross validation			External prediction	
				R^2^	RMSECV	R^2^	RMSEP	RPD
Caps	No	724.08	3.45e−04	0.92	0.73	0.77	2.24	1.20
	SNV	5.66	5.52e−03	1	0.12	0.53	1.10	1.13
	MSC	370,728	9.54e−07	0.96	0.55	0.67	2.34	0.92
	FD	16	1.95e−03	0.97	0.41	0.68	1.31	1.52
	*SD*	4	2.76e−03	0.97	0.43	0.87	0.82	2.40
	SG (7)	512	4.88e−04	0.92	0.69	0.77	2.15	1.24
	SG (15)	724.08	3.45e−04	0.91	0.75	0.77	2.29	1.19
	SNV + *SD*	0.35	5.52e−03	0.49	1.91	0.11	1.84	0.78
	FD + SG (7)	16	1.95e−03	0.97	0.41	0.67	1.32	1.51
	FD + SG (9)	16	1.95e−03	0.97	0.41	0.67	1.33	1.50
	SNV + FD+SG (9)	64	2.44e−04	0.95	0.80	0.58	1.11	1.86
Stipes	No	92,681.9	1.35e−06	0.88	1.27	0.80	3.28	1.04
	SNV	524,288	1.35e−06	0.97	0.66	0.80	3.80	0.95
	MSC	8,192	2.70e−06	0.89	1.16	0.85	2.02	1.28
	FD	32,768	1.08e−05	1	0.13	0.85	4.80	0.93
	*SD*	32,768	9.54	0.98	0.57	0.79	2.09	1.33
	SG (7)	2,896.31	4.32e−05	0.9	1.17	0.81	3.70	0.95
	SNV + FD	1,024	1.53e−05	0.94	0.82	0.85	2.65	1.20
	MSC + FD	11,585.2	1.90e−06	0.95	0.75	0.83	2.27	1.38
	FD + SG (9)	16,384	2.15e−05	1	0.13	0.86	4.61	0.96
	FD + SG (11)	1,448.15	2.16e−05	0.92	0.96	0.88	4.09	0.91
	FD + SG (13)	4,096	1.53e−05	0.96	0.72	0.89	4.25	0.94
	MSC + FD + SG (9)	5,792.62	3.81e−06	0.94	0.78	0.83	2.32	1.78

Abbreviations: FD, first‐order derivative; *SD*, second‐order derivative; MSC, multiplicative scattering correction; SNV, standard normal variate; SG, Savitzky––Golay smoothing; SG (7), Savitzky–Golay smoothing with seven points; SG (9), Savitzky–Golay smoothing with nine points; SG (11), SavitzkyGolay smoothing with eleven points; SG (13), Savitzky–Golay smoothing with thirteen points; SG (15), SavitzkyGolay smoothing with thirteen points.

## 4 CONCLUSION

The test results showed that this study did provide a fast and accurate method for species discrimination. In addition to the use of low‐level data fusion, three types of feature variables were selected simultaneously to complete mid‐level data fusion, which indicated that the PLS‐DA model based on feature variable selected by maximum Q^2^_(cum)_, GS‐SVM, and PSO‐SVM models based on low‐level data fusion had 100% discrimination accuracy allowing each mushroom classed into its real species. Moreover, this study also measured alcohol‐soluble polyphenols content in four porcini mushrooms, offered accumulation tendency of polyphenols in different parts of mushroom for the first time and predicted the polyphenol content for four porcini mushrooms. The result suggested that the way of caps data combination with second‐order derivative can be used to predict roughly polyphenols content in four porcini mushrooms. These outcomes from this work can provide academic references for origin traceability, market supervision, quality evaluation, and edible security.

## CONFLICTS OF INTEREST

The authors declare that there are no conflicts of interest regarding the publication of this paper.

## ETHICAL APPROVAL

This study does not involve any human or animal testing.
